# Bibliographical Notices

**Published:** 1855-03

**Authors:** 


*A Practical Treatise on Foreign Bodies in the Air Passages.* By J. D. Gross, M. D., Professor of Surgery in the University of Louisville, &c. With illustrations. Philadelphia: Blanchard & Lea, 1854.

It is rather surprising, considering the number of monographs which we possess on almost every subject, that no complete one has ever appeared, in any language, on foreign bodies in the air passages. The only paper mentioned by Dr. Gross as even approaching such an attempt, is the "Memoir on Bronchotomy," by Mons. Louis, published in 1759, in the Transactions of the Royal Academy of Surgery. Celebrated as this paper was in its day, few practitioners, we suspect, knew of its existence, much less were able to use it, as a work of reference, previous to its mention by our author. Several papers, it is true, have appeared in Great Britain, upon the subject; from none of these, however, can much information be obtained. A short memoir, by Dr. H. G. Jameson, of Baltimore, published in the *American Medical*

*Recorder*, is spoken of as being, for a long time, one of the best accounts of the subject in the English language. When we consider the great need of such a work, the comparative frequency of such accidents, the general ignorance of the symptoms they cause, and of the proper medical and surgical treatment to be pursued, we cannot too highly estimate our obligations to the author for the able and elaborate treatise he has given us. His zeal and industry in collecting, arranging and analysing the numerous cases, scattered through the medical journals of this and other countries, in addition to those which now, for the first time, are given to the profession, are deserving of all praise. We have read his treatise through, and we do not hesitate to say that it is in every respect a work, of which, as Americans, we have great reason to be proud.

Chapter 1st discusses the nature of those substances which enter the air passages—the alteration which they are liable to undergo from their retention—their most common situation, and finally, the mode by which they enter and are expelled.

Since the publication of Dr. Gross's treatise, two cases have been recorded which, as they belong to a class not mentioned by our author, we shall briefly mention. One of these was, the introduction, while coughing, of a plug of thick mucus into the air passages of a child, producing the usual symptoms caused by a foreign substance there; tracheotomy was performed in vain, the patient dying a short time afterwards. We record the case in the present number. The other instance alluded to* is that of a lad, eight years of age, who, while playing, was said to have been struck by one of his playfellows. When arrived at his home, a minute or so afterwards, his struggles became so violent that he could scarcely be held. When seen by Dr. Bell, a half an hour after the seizure, his countenance was livid and he was making some feeble struggles, evidently death-throes. Tracheotomy was immediately performed, and artificial respiration afterwards attempted, but without effect, as he gave but two gasps after the operation. Upon examination a diseased bronchial gland, one inch long, was discovered under the epiglottis, and extending from the rima glottidis into the larynx. It had become de-

* Medico-Chirurgical Transactions, vol. xxxvi.

tached from its bed by ulceration around it, which opened a way also for its passage into the trachea. The blow complained of was no doubt the means of forcing it from its position *into* the trachea, from which place it was driven into the glottis by strong expulsive efforts.

The case is an exceedingly interesting one, in a medico-legal point of view. Had no autopsy been made, the death would have been attributed to any other cause but the true one, and most serious consequences might have been the result to the boy who struck him, if the case had gone before a jury.

The alteration of foreign bodies in the air passages is of great practical interest to the surgeon. Though many vegetable and animal substances are liable to be occasionally softened and disintegrated by the combined action of heat, moisture and their frequent change of position in the lungs, Dr. Gross very properly reprobates, where the symptoms are severe, the absurdity of trusting to such a contingency. The place where a foreign body becomes arrested will be influenced, materially, by its size, form, and weight. When it is stopped in the larynx it may lodge either in the ventricles or between the vocal chords. It rarely is arrested in the trachea, but, having arrived at its extremity, descends most generally into the right bronchial tube. This latter circumstance is owing to the fact that the septum, at the root of the trachea, is not in the central line, but to the left of it. Hence, bodies, particularly those of considerable bulk, as they strike upon this ridge, will naturally fall over to the right side.

Most substances enter the air passages through the glottis. They may obtain entrance there, however, in many other ways; through the pharynx by ulceration, by wound of the throat, and through the walls of the chest. Cases are given of these different modes of admission.

Expulsion of foreign substances generally takes place at the glottis; occasionally, however, they are discharged through an abscess or fistula in the walls of the chest. Dr. Stanski asserts that he is acquainted with as many as twenty cases where such a result took place. The author reports an interesting case, where a sprig of a juniper tree, which had passed into the windpipe of a young child, was discharged a year afterwards, through an abscess which opened between the fifth and sixth ribs, near the nipple. The child soon began to improve, and finally recovered, with the right side of its chest much contracted.

*Chapter 2d.*—One of the immediate effects sometimes produced by the entrance of a foreign body into the larynx is sudden death. The respiration is arrested in a moment, as effectually as from a dose of prussic acid. Drunken persons, or persons laboring large under delirium tremens, it is stated, sometimes die in this way while vomiting. Disease of the epiglottis, also, renders the person having it very liable to such a termination. Occasionally, the sudden ingress of blood into the wind-pipe, during operations upon the mouth or throat, or even when tracheotomy is being performed, produces suffocation, which soon terminates in death. Two singular instances are related where blood produced this result. In one of these it poured into the wind-pipe from an ulcerated artery; in the other, the person was suffocated by a large clot from a wound of the neck pressing upon the trachea. Mechanical occlusion of the glottis, caused by the impaction of a large foreign body in the pharynx and œsophagus, is also mentioned as the occasional cause of death, two instances of which are related.

The 3d chapter treats of the pathological effects of foreign bodies. Changes in the structure of the part with which the foreign substances in contact, as well as those in its neighborhood, are stated to be the most common consequences. Occasionally, remote parts of the lung, of the trachea, and even of the larynx, become affected, either primarily or secondarily. Inflammation of the mucous membrane, mostly limited in extent, and ulceration, both of them accompanied by increase, at first, of the normal secretion and subsequently of muco-purulent matter, are mentioned as the lesions most liable to be produced. When the foreign substance is in the bronchial tubes, inflammation of one or both lungs is a very frequent result. When it has been retained for a long time, abscesses are very apt to form and continue to discharge for months and even years.

A deposit of tubercular matter, it is asserted, is sometimes induced in the tissues adjoining the foreign body. Three instances of this occurrence are related, one of which came under the author's own observation. We do not regard any of the cases as decisive of this question, as it is quite probable that the tubercular disease may have existed previously in all of them. In many instances, also, we may observe, where the foreign body was retained for years, no such result occurred.

Œdema of the larynx, pulmonary emphysema, enlargement and softening of the bronchial glands, effusions into cavity of the pleura and extensive adhesions between its opposite sides, with thickened false membranes, occur not only where the obstruction is seated in the lungs or bronchial tubes, but also, in many instances, where it has not descended below the larynx or upper part of the trachea.

The symptoms which accompany and follow the ingress of a foreign body into the air passages are discussed in Chapter 4th. These are divided by the author into those which take place at the moment of entrance, and those which arise in consequence of the sojourn of the offending substance. We have no space for an analysis of this or the following chapter, which treats of the diagnosis of foreign bodies. We can only heartily commend them to our readers as containing much instructive matter.

An account of 49 cases of spontaneous expulsion of foreign bodies, followed by recovery, is given in the 6th Chapter. In several of these instances, the substance was not expelled for years afterwards. An extraordinary case is given us where a piece of bone, swallowed by a child three years of age, remained in the lungs for sixty years. During all this period the patient was harassed with cough, profuse expectoration and other serious symptoms, and was at one time so feeble that he was compelled to remain constantly at home for eight years. At twenty-eight years of age his health began to improve, and, though suffering considerable inconvenience, he was able to work from that time forwards for fifteen or twenty years. Sixty years after the entrance of the bone, it was ejected by a violent fit of coughing, its exit being succeeded by expectoration of purulent matter streaked with blood.

Eight instances of spontaneous expulsion, followed by death, follow the above account. Five of these deaths were the consequence of induced pulmonary disease; the other three died from the effects of exhaustion, caused by the constant irritation of the offending substance.

Medical treatment, to promote the expulsion of foreign substances as well as to relieve the evil effects which their presence induce, is treated of in Chapter 7. Dr. Gross condemns the use of emetics and sternutatories, believing that much valuable time is lost in waiting for their effects. Three cases only are given where the expulsion of the foreign substance followed the action of an emetic, while 46 cases are mentioned in a table, in which no result was obtained by their exhibition. In several of these cases their action was decidedly prejudicial, and in not a few the patient came near dying from suffocation.

No patient, our author remarks, is safe as long as the foreign body remains in the wind-pipe. Should symptoms of bronchitis or pneumonia supervene, they should be treated by the ordinary remedies. The spasmodic cough, so often present, is more promptly allayed by a sup of cold water than by any other means. If the foreign body should happen to be expelled, he cautions the practitioner not to suppose all danger past, as several instances have occurred where death by pneumonia has supervened in a short time afterwards.

*Chapter* 8.—Numerous means besides operation have been resorted to, of late years, to promote the expulsion of foreign bodies from the air passages. One of these is inversion of the body. While inverted, the chest and back of the patient are repeatedly and smartly struck with the hands, or with a pillow, to dislodge the offending substance and propel it through the glottis. Several successful instances are given of this mode of procedure. The objection to it is the risk incurred from suffocation. The author advises that a preliminary opening into the trachea be made, as, without this precaution, fatal consequences may ensue. The successful cases recorded are all of heavy bodies, bullets, shot, or coins. Numerous cases are also mentioned in which this experiment was of no use.

*Chapter* 9 treats of the surgical treatment of these accidents. The only real safety in such cases, consists, according to our author, in bronchotomy. There are exceptions to the rule, but these should not affect our practice. The foreign body is frequently expelled as soon as the wind-pipe is properly opened, being often projected to a considerable distance. Occasionally, the ejection is delayed for weeks or even months after the operation, and, the wound closing, a second, and even a third operation may become necessary, several instances of which are related.

Owing to the uncertainty of the diagnosis of foreign bodies in the larynx, the author thinks that this portion of the tube should seldom be opened if it be possible to employ tracheotomy. This latter is mentioned as being a most formidable operation, particularly in children with thick, short necks. He says, "I hardly know an operation in all surgery that I would not rather undertake, than this, under such circumstances." His experience has satisfied him that chloroform greatly facilitates its performance.

Numerous and full observations concerning the proper mode of operating; how the foreign body should be extracted and what instruments should be used; the various dangers to which the patient is exposed; the dressing and after-treatment, and the mortality attendant on the operation, take up the rest of the chapter.

*Chapters* 10, 11 and 12, are filled up with tables and descriptions of cases of laryngotomy, tracheotomy, and laryngo-tracheotomy. 13 cases of the former, 60 of tracheotomy, and 10 of laryngo-tracheotomy are related, where the expulsion of the foreign body was followed by recovery. 4 cases of laryngotomy, 8 of tracheotomy, and 3 of laryngo-tracheotomy were followed by the death of the patient.

In *chapter* 13, three cases are related where the operation of bronchotomy was performed in two instances twice, and in the third, three times. Two of these cases terminated fatally.

In *chapter* 14, several cases of tracheotomy are given, in which no foreign body was found. These cases, it is thought, are much more numerous than our records would lead us to suppose, as many surgeons are very averse to publishing their unsuccessful cases.

*Chapter* 15 is devoted to the consideration of cases of death without operation, and without expulsion of the foreign body. 21 of these cases are related; many, if not all of them, might have recovered under an operation.

Tracheotomy has been occasionally performed on the inferior animals, instances of which are given in chapter 16. The author regrets that such a proceeding is not more frequently resorted to.

A general summary of the whole subject, containing the conclusions which may be fully and legitimately deduced from a consideration of what he has given us, arranged under diagnostic, pathological, therapeutic and operative heads, occupies the last chapter.

The field of enquiry taken up by Dr. Gross, has been hitherto so much neglected, that the thanks of the profession are largely due him for the zeal and care which he has expended on its cultivation. His observations, throughout, are characterized by sound sense, and will still further enhance his reputation as a most judicious and instructive writer. We earnestly recommend its perusal to the profession. The work is courteously and deservedly dedicated to Dr. Norris, of Philadelphia.

---

*Puerperal Fever as a Private Pestilence.* By OLIVER WENDELL HOLMES, M. D., Parkman Professor of Anatomy and Physiology in Harvard University. Boston: Ticknor & Fields, 1855.

"This essay," says the author in his introduction to the republication, "was read before the Boston Society for Medical Improvement, and at the request of the Society, printed in the New England Quarterly Journal of Medicine and Surgery for April, 1843. As this journal never obtained a large circulation, and ceased to be published after a year's existence, and as the few copies I had struck off separately were soon lost sight of among the friends to whom they were sent, the essay can hardly be said to have been fully brought before the profession."

To Dr. Meigs' lately published work on puerperal diseases, we are indebted for the republication of this excellent and useful pamphlet. The author, with an industry and zeal worthy of all commendation, has collected and arranged a mass of evidence in favor of the sometimes contagious nature of puerperal fever, which we believe and hope will go far towards acting as an antidote to the baneful teachings and theories of the Jefferson and University Professors of Obstetrics, upon this subject. The opinions of Drs. Meigs and Hodge, as individual practitioners, might with propriety pass without challenge or remonstrance, but in their capacity of teachers, lecturing as they do, annually, to upwards of 1000 students, and thereby promulgating their doctrines, however unsustained, to every section of this vast country, they rise to an importance which renders it the bounden duty of every man, who thinks otherwise, to speak his antagonism boldly, however unpalatable to cliques and coteries, and make it heard in every quarter of the land.

Dr. Wendell Holmes has nobly fulfilled this obligation. He has collected a mass of evidence on the subject which cannot be slighted; he has arranged his materials admirably, and followed the statements up by a course of clear logical reasoning, not the less acceptable for being expressed in a polished, scholarly style. The thanks of the profession are due to this gentleman for the re-publication of this brief work; its moral tone and right honest expression of opinion are alike honorable to its author, and elevating to the profession, which is bound thoroughly to weigh its testimony before rejection. Were it a mere jumble of unsupported theories, or had the author, after the fashion of some of his contemporaries, made his facts for the especial convenience of a theory, its consignment to an eternal repose, with many of its generation, would have been strictly just; but sustained as it is, by a long array of illustrious authorities, and by clear logical deduction, sarcasm is futile, and contempt entirely misplaced. Dr. Wendell Holmes carries heavy metal, which will make itself heard and respected even by those great guns that, in full target practice, matutinally explode to five hundred promising young gentlemen.

We do not propose to review Dr. Holmes' pamphlet systematically, or even to indulge largely in quotations; it must be read to be fully appreciated. The following passage, however, so perfectly coincides with our own sense of right, that we cannot resist the inclination to favor our readers with it.

"If the physician does not at once act on any reasonable suspicion of his being the medium of transfer, the families where he is engaged, if they are allowed to know the facts, should decline his services for the time. His feelings, however interesting to himself, should not be even named in this connection.

A physician who talks about *ceremony* and *gratitude*, and *services rendered* and the *treatment he got*, surely forgets himself; it is impossible that he should think of these small matters, where there is even a question whether he may not carry disease and death and bereavement into every one of 'his families,' as they are sometimes called."

The original, as it appeared in the New England Quarterly Journal, has not been altered in a single syllable, but some twelve pages of an excellently written introduction have been published along with it, part of which is devoted to Dr. Meigs and his book on Puerperal Diseases, and part to a discussion on Contagion ; both subjects are artistically handled. The following passage, in which the author alludes to the cavalier mode in which Dr. Meigs flattered himself he had put him out, is a good specimen of his style.

"One unpalatable expression I suppose the laws of construction oblige me to appropriate to myself, as my reward for a certain amount of labor bestowed on the investigation of a very important question of evidence, and a statement of my own practical conclusions. I take no offence and attempt no retort. No man makes a quarrel with me over the counterpane that covers a mother with her new born infant at her breast! There is no epithet, in the vocabulary of slight and sarcasm, that can reach my personal sensibilities in such a controversy. Only just so far as a disrespectful phrase may turn the student aside from the examination of the evidence, by discrediting or dishonoring the witness, does it call for any word of notice."

Coincidence is the resource of those gentlemen who maintain that puerperal fever cannot be conveyed to its victims by the nurse or attending physician. Of course, Dr. H. has no difficulty in showing the utter weakness of such a position ; and in reply to the argument of Dr. Meigs, that a contagious disease needs time for incubation, and that the sudden appearance of the dangerous symptoms in child-bed fever preclude the possibility of its being caused by contagion, mentions numerous instances of the fearful suddenness of some contagious visitations ; where the patients were struck down by disease not an hour after having come for the first time in contact with its cause. Instances might have been cited where the germs of the same malady (typhus) have lain dormant in the system for many days. Who can explain the reason of this difference ? The bite of the rattlesnake acts almost instantaneously, and has destroyed life in three hours, while the virus of hydrophobia will incubate, as it called, for months. Can all the medical colleges put together tell us why ? In the face of such strange variety and seeming contradiction who can pretend, by analogy, to satisfy himself of what contagion is, or what it is not ?

Our author says :

"If the medical theorist insists on being consulted, and we see fit to indulge him, he cannot be allowed to assume that the alleged laws of contagion, *deduced from observation* in other diseases, shall be cited to disprove the alleged laws *deduced from observation* in this."

Again, speaking with reference to the communication of the disease by third parties:

"It is not necessary to consult any medical theorist as to whether or not it is consistent with his preconceived notions that such a mode of transfer should exist."

These remarks are such self-evident truths that they require no comment.

Dr. Wendell Holmes closes his introduction with the following remarks:

"I trust that I have made the issue perfectly distinct and intelligible; and let it be remembered that this is no subject to be smoothed over by nicely adjusted phrases of half assent and half censure divided between the parties. The balance must be struck boldly, and the result declared plainly. If I have been hasty, presumptuous, ill informed, illogical; if my array of facts means nothing; if there is no reason for any caution in the view of these facts; let me be told so, on such authority that I must believe it, and I will be silent henceforth, recognizing that my mind is in a state of disorganization. If the doctrine I have maintained is a mournful truth; if to disbelieve it, and to practise on this disbelief, and to teach others so to disbelieve and practise, is to carry desolation and to charter others to carry it into confiding families, let it be proclaimed as plainly what is to be thought of the teachings of those who sneer at the alleged dangers, and scout the very idea of precaution. Let it be remembered that *persons* are nothing in this matter; better that twenty pamphleteers should be silenced, or as many professors unseated, than that one mother's life should be taken. There is no quarrel here between men, but there is deadly incompatibility and exterminating warfare between doctrines. *Coincidences* meaning nothing, though a man have a monopoly of the disease for weeks or months; or *cause and effect*, the cause being in some way connected with the person; this is the question. If I am wrong, let me be put down by such a rebuke as no rash declaimer has received since there has been a public opinion in the medical profession of America; if I am right let doctrines which lead to professional homicide be no longer taught from the chairs of those two great Institutions. Indifference will not do here; our Journalists and Committees have no right to take up their pages with minute anatomy and tediously detailed cases, while it is a question whether or not the 'black death' of child-bed is to be scattered broadcast by the agency of the mother's friend and adviser. Let the men who mould opinions look to it; if there is any voluntary blindness, any interested oversight, any culpable negligence, even, in such a matter, and the facts shall reach the public ear; the pestilence carrier of the lying-in chamber must look to God for pardon, for man will never forgive him."

*An Inquiry into the Pathological Importance of Ulceration of the Os Uteri, being the Croonian Lectures for the year* 1854. By CHAS. WEST, M. D., &c., author of "Lectures on the diseases of Childhood and Infancy." Philadelphia, Blanchard & Lea, 1854.

The opinion that ulceration of the os uteri is the first in a succession of processes which occasion uterine pain, menstrual disorder, leucorrhœa and other affections of the generative system, being a question on which the profession was much divided, induced our author to put the matter to the test of a rigid inquiry, that he might establish, if possible, its truth or error. The numerous arguments, analogies and facts brought to bear upon the discussion of this question, are arranged under four principal heads, as follows:

"In the *first place,* we may inquire how far these statements receive confirmation from what we know of the anatomy and physiology of the uterus in a state of health.

Still, what answer soever we may receive to this question, it cannot, from its very nature, be conclusive; it may render a certain occurrence probable or improbable, may substantiate or disprove the correctness of certain opinions or explanations, but cannot invalidate the evidence of positive facts.

In the *second place,* we may try to ascertain whether examination of the dead body shows the morbid conditions of the os uteri which have been described to be frequent or rare, slight or extensive; and we may also endeavor to make out what connection subsists between ulceration of the mucous membrane of the os and cervix uteri, and other changes in the tissue of the organ.

It must, however, be borne in mind that many evidences of disease, such as are very obvious during life, may be greatly obscured, or may even entirely disappear after death: and further, that uterine disorders of the class which we are considering, though exceedingly painful, and seriously interfering with a woman's health and comfort, are yet not of a kind to prove the direct occasion of her death. Evidence derived from this source will therefore be open to the objection that it understates both the frequency and the importance of these diseases.

We may inquire, in the *third place,* whether there is any condition in which ulceration of the os uteri comes under our notice unconnected with other disease, and with such circumstances as to admit readily of our observing its character and watching its course and consequences. Such a state of things presents itself to us often in the case of the procident uterus, since the irritation to which the displaced organ is unavoidably exposed has the almost invariable effect of producing ulceration of the surface of the os uteri, and the immediately adjacent parts of the organ.

But, whatever conclusions we may deduce from this source they are open to all the objections inseparable from analogical reasoning. The probabilities of certain occurrences taking place in the uterus under other circumstances may be increased or weakened; but the evidence still falls short of absolute proof, either of the affirmative or of the negative of the question.

The *fourth* and most important inquiry of all concerns the frequency of these ulcerations of the os uteri under those circumstances in which they ordinarily come under our notice, and call, or are supposed to call, for our interference. This inquiry, however, must include not only the frequency of ulceration, but also the conditions generally associated with it, and the symptoms to which it commonly gives rise. If the alleged symptoms of ulceration are found to be not rarely present without ulceration is discovered, even where there are no symptoms; or if, in the same case, the ulceration may vary in extent, with no corresponding change in the symptoms; if an indurated state of the cervix exists without ulceration, and ulceration, even of long standing, without induration—the conclusion, especially if supported by the answers obtained to our previous inquiries, seems to me irresistible, that the importance of inflammation of the cervix and of ulceration of the os uteri has been overstated; that they are not the cause of all the symptoms which they have been alleged to occasion, and that, in the treatment of uterine disease, many other considerations must influence us more than the mere removal of ulceration of the orifice of the womb.

If this were proved, it would still remain for us to consider whether, in any case, we may fairly look upon the ulceration of the os uteri as a symptom calling for distinct recognition and special treatment. There are, I am aware, some persons of deserved repute who will look upon this inquiry as superfluous; but, for my own part, I do not conceive that, even if we arrived at a conclusion never so unfavorable to the supposed great importance of ulceration of the os uteri, we should be thereby entitled to regard its symptoms as a mere delusion, its very existence as little more than a figment of the fancy."

The discussion of the above questions occupies the first two lectures. The third lecture takes up the various causes of uterine ailments. These are shown to be frequently independent of local disease, as in the case of chlorosis, of hepatic disorder, of granular disease of the kidneys, or where the affection is dependent upon the rheumatic or gouty diathesis; ulceration of the os uteri, when present in such cases, as well as in those where disease begins in the uterus itself, as in the affections following pregnancy, abortion, delivery, &c., being shown to be of very slight and secondary importance.

To meet the objections which might reasonably be urged against the above opinions, viz.: that experience has proved that the application of caustic to the os uteri has been followed by very successful results, and that, too, where relief has been vainly sought by other methods, Dr. West suggests numerous explanations, for which we must refer our readers to the work itself.

The injurious effects resulting from the doctrine and practice so ably combatted by Dr. West, are undoubtedly great. We firmly agree with the author that the belief in such a condition as the cause of so many and important mischiefs, is calculated, in fact, must naturally tend to relax the efforts of the practitioner to investigate closely these different affections and their consequences. "He unlearns," as Dr. West very truly remarks, "what physiology might teach him of the uterus and its functions, and sees in all its various manifestations of disorder, the expression of one fact, and one fact alone; namely, the existence of ulceration of its womb and its reactions, first on the uterine system, then on the general health." The very general acceptance of Dr. Bennett's views by the profession, has thus produced a routine practice in these diseases, which has much retarded the progress of true science. Nor should such a result be wondered at; the very simplicity of the pathology inculcated and the easy application of the remedy, involuntarily and forcibly commending themselves to every one desirous of obtaining a ready solution of these often intricate affections.

Nor should we forget to take into account the physiological reaction of the doctrines spoken of upon the susceptible temperament of woman. So important to the mind of almost every female, arrived at the age of discretion, is the rôle played by her sexual system, in the production of disease, that she readily becomes an adherent to the idea that ulceration of the womb is the source of all her uterine troubles. Sinbad's old man of the sea clung not more tenaciously nor more oppressively to his victim, than does this hateful incubus to her disordered imagination, when she is once possessed by it. The mischievous results of such an engrossing, absorbing idea are so admirably and strongly depicted by our author that it must, hereafter, be impossible to ignore its importance, both in the confirmation and augmentation of her disease.

The innate and thorough loathing with which every right-minded woman must regard the introduction of a speculum into her vagina, with all its attendant mortifications, is another very serious objection to such a proceeding. No lower deep of degradation can well be imagined by a young and innocent mind, than that of having her person thus exposed and commented upon by others. Every instinct of her nature must revolt at such an outrage. Nothing but the most positive necessity can be pleaded as the practitioner's justification for such an act. That it is frequently done, however, where there is no such necessity, we have not a shadow of a doubt. We are happy, under such circumstances, to have such an authority as that of our author, in stating "that the importance of inflammation of the cervix, and of ulceration of the os uteri has been overstated; that they are not the cause of all the symptoms which they have been alleged to occasion; and that, in the treatment of uterine disease, many other considerations must influence us more than the mere removal of ulceration of the orifice of the womb."

The style and language of the lectures are fully worthy of the high reputation of the author. Though discussing a question on which there has been much intemperate expression of opinion, their tone is calm, courteous and gentlemanly. We cannot recal to mind, in fact, any controversial work, which, in this respect, is more deserving of imitation. With the most unfeigned respect for the talents and high character of the author, we sincerely wish for his Croonian Lectures, what they so richly deserve, an extensive circulation.

---

*Report of the Select Committee of the Senate of the United States on the sickness and mortality on board Emigrant Ships.* Washington: Beverly Tucker, Senate Printer. 1854.

The Report under consideration is one of grave significance, showing as it does that a large amount of human life is annually sacrificed by the ignorance or cupidity of ship-owners on both sides of the Atlantic. Of no more importance in the eyes of the shipper and his employés than a bale of merchandize, the poor emigrant, frequently predisposed to disease by sorrow and privation, is remorselessly thrust into a space, the cubic dimensions of which, in many instances, hardly exceed that alloted to the dead, where the slightest modicum of pure air never enters, and with just sufficient light to render darkness visible. Thus "cribbed, cabined and confined" in their sub-aqueous abode,* eating scanty and half-cooked rations, the exhalations from their lungs and skin, and too often the more offensive excretions of their bodies accumulating around them, it does not surprise us to learn "that of those who embark for an Atlantic voyage on any one of a certain class of ships, one in every twelve of them but steps into a coffin, and nearly nine per cent. will either never reach the promised land or will die soon after."

The diseases which thus almost deciminate the steerage passengers *in transitu* between the eastern and western shores of the Atlantic, are those terrible zymotic scourges, typhus fever, cholera and small pox; typhus being the one to which the emigrant is most liable. If the influences which originate these maladies, or form the chief fuel for their devastating flames, were beyond the ken of the profession, then could we only mourn the impotency of our science; but it being notorious that typhus fever acknowledges no other habitat, and that cholera and small pox commit their most frightful ravages among those subjected to the hygienic privations we have declared to exist in many emigrant ships, we cannot but express our unqualified censure of the owners of such vessels, who, for the sake of a few dollars, would thus consign nine in every hundred of their fellow men to certain death.

That enlightened legislation can easily check this undue mortality it is needless to say, and it certainly is the duty of physicians to urge it. At least 250 cubic feet of space should be allotted to each adult passenger, and a thorough ventilation, and strict cleanliness of person and berth enforced. Both sexes should be compelled to remain as much as possible "on deck," and the beds and bedding should be daily exposed to the open air. The food should be cooked *for*, not *by*, the passengers, and its quality be better than the present law requires. The

*Many passenger ships have three decks, the lowest of which, or the "orlop deck," lies immediately above the kelson, and is thus completely sub-aqueous.

services of a qualified physician we consider indispensable, and he should be assisted by at least two experienced nurses. These are the chief sanitary regulations necessary for the object in view, and as they are not only practicable but of easy application, we earnestly trust that the government of the United States will promptly enact such laws as will enforce their universal observance.

---

*What to Observe at the Bed-Side, and after Death, in Medical Cases. Published under the authority of the London Medical Society of Observation. Second American, from the Second and enlarged London Edition.* Philadelphia, Blanchard & Lea.

We are glad to see that the above most useful and complete work—which should, bye the bye, be in the hand of every practitioner in our country—has reached a second edition. It is creditable to the Profession to find such a book properly appreciated.

The puplishers deserve praise for the excellent style in which it is issued. Its paper and type are unexceptionable.

---

*Principles of Physiology; designed for the use of Schools, Academies, Colleges and the general reader. Also, an essay on the Preservation of Health, with fourteen quarto plates, and over eighty engravings on wood, making in all nearly two hundred figures.* By J. C. Comstock, and B. N. Comings, M. D. New York, S. S. & W. Wood, 1855.

The above work is well adapted to the use of the general student. It will be found to present clear outlines of a subject that should be an essential branch of every one's education.

